# A Quantitative Live-Cell Superresolution Imaging Framework for Measuring the Mobility of Single Molecules at Sites of Virus Assembly

**DOI:** 10.3390/pathogens9110972

**Published:** 2020-11-21

**Authors:** Nicholas S. Groves, Merissa M. Bruns, Schuyler B. van Engelenburg

**Affiliations:** Molecular and Cellular Biophysics Program, Department of Biological Sciences, University of Denver, Denver, CO 80210, USA; nicholas.groves@du.edu (N.S.G.); merissa.bruns@du.edu (M.M.B.)

**Keywords:** HIV-1, virus assembly, Env, Gag, single particle tracking, superresolution

## Abstract

The insurgence of superresolution microscopy into the fields of virology and microbiology has begun to enable the mapping of molecular assemblies critical for host–pathogen interfaces that organize on a scale below the resolution limit of the light microscope. It is, however, challenging to completely understand the molecular interactions between host and pathogen from strictly time-invariant observations. Herein, we describe a method using simultaneous dual-color superresolution microscopy to gain both structural and dynamic information about HIV-1 assembly. Specifically, we demonstrate the reconstruction of single virus assembly sites using live-cell photo-activated localization microscopy (PALM) while concurrently assessing the sub-viral mobility of the HIV-1 envelope glycoprotein during interaction with the viral lattice. We propose that our method is broadly applicable to elucidating pathogen and host protein–protein interactions through quantification of the dynamics of these proteins at the nanoscale.

## 1. Introduction

Since its inception, superresolution microscopy has been broadly adapted to many fields of biological study [1,2,3,4,5]. Such methods offer molecular specificity and resolution improvements up to 10-fold over conventional diffraction-limited microscopy, enabling the reconstruction of biological assemblies approaching tens of nanometers [6]. With these tools in hand, researchers have been able to assess cellular spatial organization of molecular assemblies created by pathogens, enabling the nanoscale visualization of host–pathogen interfaces [7,8,9,10,11,12,13,14]. In particular, superresolution imaging has provided powerful insight into the mechanisms of human immunodeficiency virus-1 (HIV-1) particle biogenesis and maturation [6,15,16,17,18,19,20,21,22,23,24]. While these studies have been powerful for uncovering new aspects of protein organization during HIV-1 biogenesis, this methodology has been largely relegated to time-independent measurements and lacks spatiotemporal information regarding viral protein coalescence. These shortcomings have been addressed using fluorescence recovery after photobleaching (FRAP) to measure bulk diffusion rates for viral molecules, however, this technique cannot provide resolution below the the diffraction-limit [25]. Stimulated emission depletion microscopy in conjunction with scanning fluorescence correlation spectroscopy (STED-FCS) presents a route to measure diffusion rates of viral molecules below the resolution limit of the light microscope [26]. In addition, single particle tracking (SPT) is an orthogonal and readily accessible method that can provide spatiotemporal information of single viral molecule dynamics on the scale of one to tens of nanometers [27]. Combinations of both STED and SPT have also been demonstrated [28].

Upon infection, HIV-1 Gag oligomerizes on the inner leaflet of the plasma membrane in T-cells by a cooperative interaction between its N-terminal membrane-binding domain and internal Capsid (CA) oligomerization domain [29,30,31,32,33]. After reaching a critical mass of oligomers, the forming lattice will buckle the plasma membrane to create a virus bud [34,35]. In order to form an infectious particle, the HIV-1 envelope glycoprotein (Env) must traffic from the biosynthetic pathway to reach the plasma membrane. Upon reaching the plasma membrane, Env freely diffuses and either becomes trapped in a newly formed Gag lattice or is endocytosed [36,37]. Previously, we described the spatiotemporal dynamics of HIV-1 Env on a sub-viral scale (tens of nanometers) during HIV-1 biogenesis on living cells [27]. Our SPT method allowed for simultaneous measurement of heterogeneous diffusion modalities and trapping of Env at sites of Gag lattice formation, which was dependent upon residues in both Gag and Env. Our previous approach relied on conservative estimates of Env proximity to Gag lattices due to diffraction-limited resolution of virus assembly sites, creating uncertainty in the location of the perimeter of these assembly sites. For strong phenotypic differences this uncertainty becomes negligible, however, this error becomes significant as genetic phenotypes become more nuanced. Herein, we expand on our previous methodology by providing a framework for measuring live-cell nanoscale dynamics using superresolution photo-activated localization microscopy (PALM) of the Gag lattice paired with single particle tracking of HIV-1 Env. Although we have focused this methodology on HIV-1 assembly, this method is broadly applicable to other virus species and microbial receptor interactions.

## 2. Results

### 2.1. Simultaneous Superresolution Reconstruction and Tracking of HIV-1 Gag and Env

**Figure 1:**
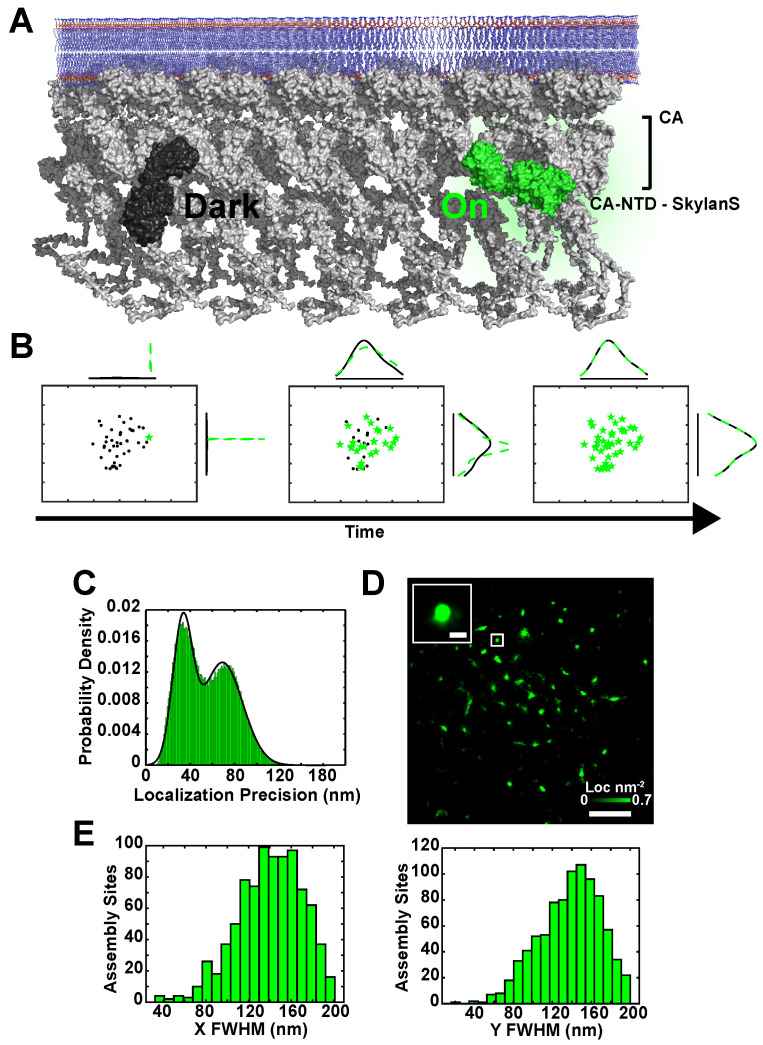
CA-Skylan-S probes accurately reconstruct HIV-1 assembly sites on live infected T-cells. (**A**) The HIV-1 Gag lattice (Gray) oligomerizes on the inner leaflet of the plasma membrane (Blue). The genetically encoded anti-CA nanobody fused to Skylan-S (CA-Skylan-S) binds specifically to the N-terminal domain of Capsid. Skylan-S undergoes photoswitching upon illumination with blue (473–488 nm) laser light, moving single Skylan-S molecules between a ‘Dark’ and ‘On’ fluorescent state (black and green, respectively). (**B**) Example of localization accumulations for a single diffraction-limited virus assembly site on a live infected CEM-A cell expressing CA-Skylan-S probes. Green represents ‘On’ molecular localizations accumulated over time. Black represents the final accumulation of localizations for the reconstructed virus assembly site. Curves are the respective probability densities in horizontal and vertical directions (X,Y) for the superresolution reconstruction showing a Gaussian-like distribution after sufficient sampling of lattice-associated CA-Skylan-S probes. (**C**) Aggregate of all uncertainties in position for each localization in all data sets is demonstrated (σ). The histogram was fit to a bimodal Gaussian probability density ($\mu_{\sigma_{low}}=32.9\pm 8.9,\mu_{\sigma_{high}}=68.9\pm 18.2$ nm). The larger peak was eliminated by filtering localizations to σ<40 nm and likely represents localization events in areas of high cellular background. (**D**) Assembly sites on the surface of live CEM-A T-cells were reconstructed using the localization centroids with their respective uncertainties (scale: Image = 5 μm, Inset = 200 nm. Maximum localization density: 0.7 localizations per nm${}^{2}$). (**E**) Full-width at half-maximum (FWHM) for segmented virus assembly sites (normal distribution fit: $\mu_{X_{FWHM}}=139.8\pm 30.5$ nm; $\mu_{Y_{FWHM}}=137.7\pm 31.3$ nm; fit not shown). FWHM in both coordinates converge on the theoretical size of HIV-1 particles, demonstrating the ability of CA-Skylan-S probes to superresolve sites of virus assembly on living infected T-cells.

To track HIV-1 Env with respect to sites of virus assembly on the plasma membrane of living cells, we used total internal reflection fluorescence microscopy (TIRF-M) with simultaneous dual-wavelength monitoring. To reconstruct sites of Gag assembly below the diffraction-limit of the light microscope, we used a genetically encoded Camelid antibody (nanobody), targeting the Capsid (CA) domain of Gag, fused to the photoswitchable fluorescent protein Skylan-S [38,39], and expressed from the viral genome. Previously, we demonstrated that the CA nanobody has no known perturbing effects on the formation of virus particles [27]. We screened several green photoswtichable fluorescent protein variants from the Skylan series and selected Skylan-S (mEOS3.1 parent with H62S mutation) as it displayed ideal photoswitching rates and photon output for our desired frame rate. Unlike mEOS3.1, Skylan-S does not undergo photoconversion from a green to red form [39], instead it transitions between ‘on’ and ‘off’ green fluorescent states (Figure 1A).

Specifically, these single fluorescence events are captured during high-speed streaming and can be subsequently fit to a Gaussian function to determine the position of the fluorescent probe. This centroid and the number of integrated photons are used to determine the uncertainty in the position measurement [1] (Figure 1B,C). The accumulation of these points and their uncertainties in time enables the reconstruction of the Gag lattice below the diffraction-limit using the live-cell PALM method [40] (Figure 1D and Figure S1). To determine whether assembly sites were reconstructed to the approximate size of an HIV-1 particle in 2-D cartesian coordinates, they were fit to a normal distribution and full-width at half maximum (FWHM) was calculated. The approximate size of the HIV-1 virus particle is ≈145 nm [41], which validates 138 ± 31 nm as our mean FWHM of assembly sites (Figure 1E). We found that membrane movement and microscope drift were negligible during rapid acquisition (100 frames per second (fps); 30 s interval) as FWHM reconstructions between x and y dimensions varied by 5.8 nm (4%) for each assembly site measured (Figure S2). Importantly, simulations of our experimental system suggest that our FWHM reconstructions of virus assembly sites results from the localization of more than one CA-Skylan-S molecule (Figure S3).

**Figure 2:**
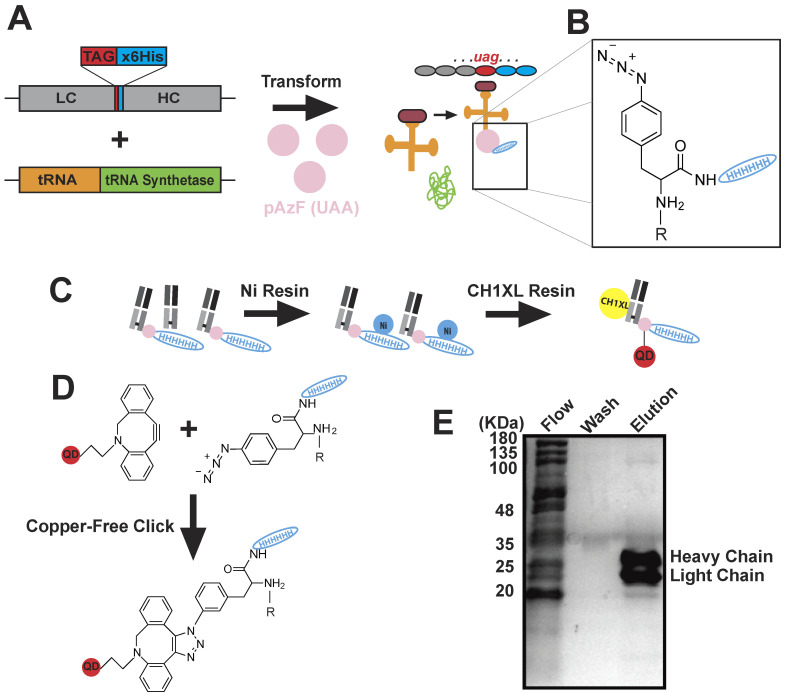
BG18-QD625 design and production. (**A**) Recombinant bacterial expression system for production of BG18 containing an unnatural amino acid (UAA). E. Coli strains selected for both pCOMB3H-BG18-Amber fab and H-4-azido-phenylalanine(pAzF) tRNA/ tRNA-synthetase expression plasmids produce UAA-incorporated light chain BG18 upon pAzF-charged tRNA anticodon (AC) recognition of the Amber stop codon. (**B**) The incorporation of the UAA allows for noncanonical expression of p-azido-L-phenylalanine thereby, providing a site for a quantum dot click reaction as well as a His-(×6) tag for purification of UAA-incorporated fab (R = BG18 fab). (**C**) Cartoon protocol for the purification of BG18-QD625: (1) Metal-ion affinity chromatography of Amber-suppressed BG18 fab possessing a hexahistidine repeat after the pAzF UAA, (2) purified BG18-pAzF fab is then conjugated to DIBO-QD625 using copper-free click chemistry, (3) unreacted DIBO-QD625 is subsequently removed from BG18 fab by affinity chromatography using anti-human IgG CH1-domain resin, (4) unlabelled BG18-pAzF fab is then removed from the BG18-QD625 conjugates using a 100 kDa molecular-weight cutoff filter. (**D**) Molecular cartoon of the BG18-QD625 conjugation strategy using the copper-free click chemistry reaction. The covalent coupling reaction occurs between the strained alkyne group and the azide moiety. (**E**) SDS-PAGE of flowthrough, wash, and elution fractions after BG18 purification from both metal-ion and CH1XL affinity chromatography. Heavy and light chains of fab complexes are purified to near homogeneity in stoichiometric abundance as assessed by Coomassie R-250 staining.

**Figure 3:**
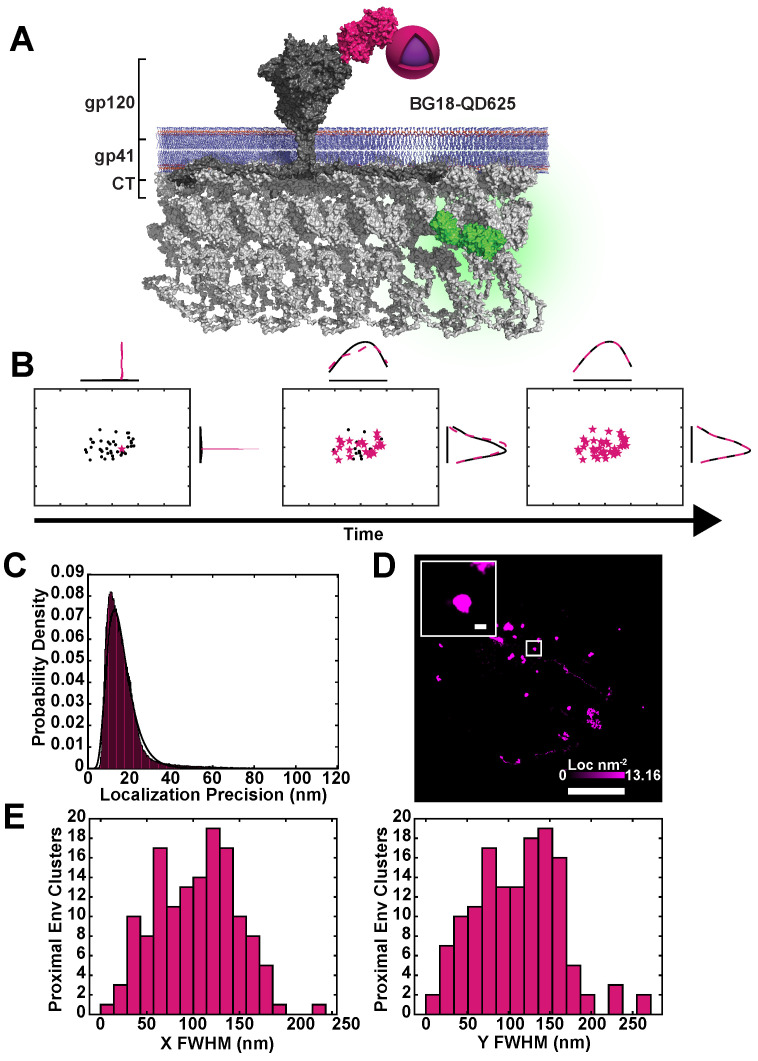
BG18-QD625 is a highly specific monovalent probe for high-density localization of single HIV-1 Env trimers. (**A**) HIV-1 Env diffuses freely on the plasma membrane until encountering a Gag lattice. BG18-QD625 (Magenta) binds to a glycopeptide (V3) on the HIV-1 Env gp120 ectodomain. The gp120 domain non-covalently associates with the ectodomain of gp41, which is anchored to the membrane through the transmembrane and Cytoplasmic Tail (CT) domains, with the CT putatively interacting with or sterically trapped by the Gag lattice. (**B**) Temporal point localization of a single Env trimer reconstructs a diffusion trajectory. Magenta stars represent new localizations of a single BG18-QD625 probe per frame. Black represents the entire accumulation of localizations over the time of acquisition. Curves represent the probability densities in horizontal and vertical (X,Y) dimensions for sub-diffraction limited distributions of mobile trimers labeled by BG18-QD625. (**C**) The localization precision of BG18-QD625 labeled Env trimers is σ=16.1±6.6 nm, equating to roughly 10% of the diameter of a single virus particle, allowing for mobility measurements at a sub-viral scale. (**D**) Tracking of single Env trimers on living cells demonstrates that Env has a range of mobility distributions. The maximum density of localizations is 13.2 localizations per nm${}^{2}$ (magenta color bar) over 30 s of sampling. Inset shows a highly confined Env trimer displaying an apparent normal distribution of displacements (inset scale bar is 200 nm; full image scale bar is 5 μm). (**E**) Single molecule trajectories of Env classified as proximal to sites of assembly were fit to a normal curve and the FWHM was calculated (fits not shown). These tracks proved to be highly confined at the sub-viral level ($\mu_{X_{FWHM}}=103.3\pm 43.2\mathrm{nm}$ and $\mu_{Y_{FWHM}}=111.0\pm 52.2$ nm). Clusters of Env localizations were found to be 30% smaller than the average size of virus assembly sites. This suggests that Env may be relegated to a sub-region of the viral lattice.

To visualize single Env trimers diffusing proximal and incorporating into Gag assembly sites, we labeled Env trimers with a modified anti-Env antibody fragment (fab) conjugated to a quantum dot. The anti-Env fab, BG18, has nanomolar affinity for the glycan-V3 region of the gp120 ectodomain of Env [42]. The fab was produced recombinantly in bacteria in order to incorporate an unnatural amino acid, p-azido-L-phenylalanine, capable of reacting site-specifically to a dibenzocyclooctynol (DIBO)-modified quantum dot via copper-free click chemistry [27] (Figure 2 and Figure 3A).

Conjugation of the fab to Quantum Dot 625 (QD625) provided a mean localization precision of 16.1 ± 6.6 nm at 100 fps when bound to surface-exposed Env (Figure 3C). With high spatiotemporal resolution and sparse cellular labeling of Env using the BG18-QD625 probe, we were able to visualize and quantify sub-viral confinement of Env within single reconstructed Gag lattices (Figure 1D and Figure 3B). No significant cross-talk between the two fluorescent channels was observed (Figure S4) and QD625 fluorescence intensity remained relatively stable during imaging intervals (Figure S5). Env in proximity to Gag centroids were fit to a normal distribution to asses the FWHM of confinement. The mean FWHM of Env positional measurements within the viral lattice was 101 ± 48 nm, corresponding to 73 percent of the average size of viral assembly sites measured by live-cell PALM (Figure 3E).

### 2.2. Sub-Viral Quantification of Env Diffusion

**Figure 4:**
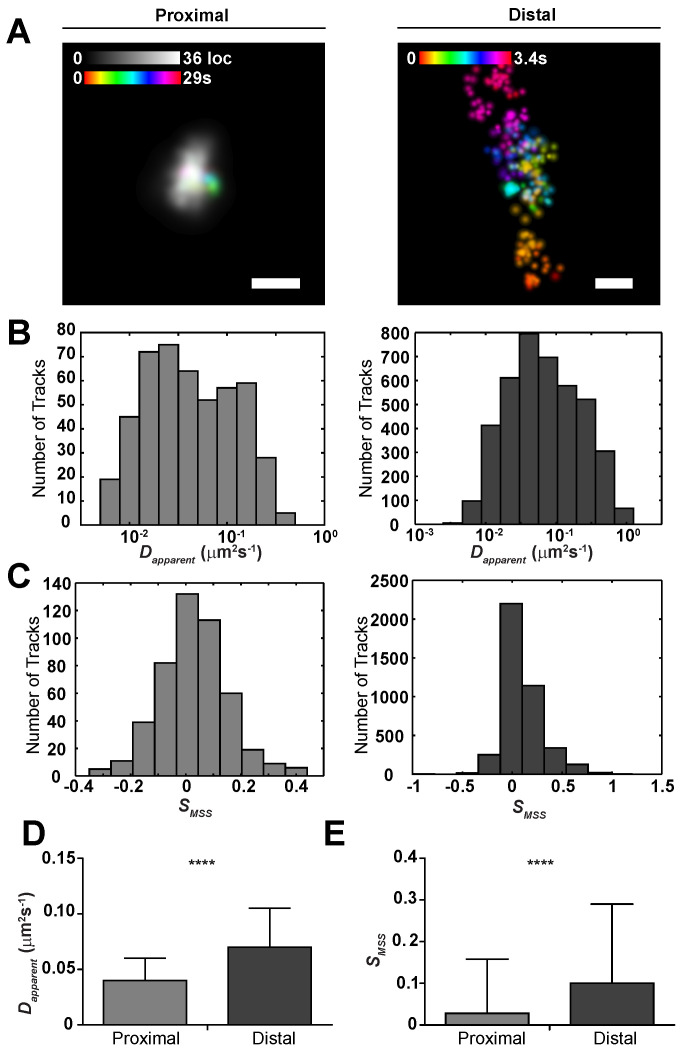
HIV-1 Env is highly confined at sites of assembly and diffuses freely when non-proximal to the Gag lattice on the surface of infected CEM-A T-cells. (**A**) Representative examples of Env diffusion ‘Proximal’ and ‘Distal’ to sites of assembly (scale bars are 200 nm; Env: time-colored gradient; Gag: gray localization density). When proximal, Env was never observed to escape the Gag lattice over the sampling period, yet is able to freely diffuse when distal to assembly sites. Width of points represent uncertainty in localization of a single molecule in the time-series. (**B**) Histograms of the apparent diffusion coefficient ($D_{apparent}$) for proximal and distal tracks in log${}_{10}$ scale ($D_{apparent}=0.04\pm 0.02$ μm${}^{2}$× s${}^{-1}$ and 0.07±0.04 μm${}^{2}\times$s${}^{-1}$, respectively). (**C**) Histograms of the slope of the moment scaling spectrum ($S_{MSS}$) for proximal and distal tracks ($S_{MSS}=0.03\pm 0.13$ and 0.10±0.02, respectively). (**D**,**E**) $D_{apparent}$ and $S_{MSS}$ for proximal versus distal trajectories were found to be statistically significant with a respective increase in both parameters for distal trajectories suggesting that Env trimers are less mobile and more sub-diffusive when proximal to a Gag lattice. Significance was assessed using a two-tailed unpaired *t*-test ($P_{D_{apparent}}=2.67\times 10^{-17}$; $P_{S_{MSS}}=1.04\times 10^{-15}$; α=0.0001; $N_{Proximal}=476$, $N_{Distal}=4089$). Error bars represent standard deviation.

With our results suggesting that single Env trimers in proximity to the Gag lattice have some sub-viral mobility, we then sought to quantify the differences in mobility between Gag lattice proximal and distal Env trimers. Trimers were deemed proximal to a Gag lattice if they were found within a radius of influence (ROI) measured from the centroid of the Gag lattice by the following equation:

(1) $$ROI=\frac{\sqrt{FWHM_{X}^{2}+FWHM_{Y}^{2}}+2\sigma_{FWHM}+\sigma {}_{Registration}}{2}$$

where FWHM-X/-Y are the virus assembly site dimensions, $\sigma_{FWHM}$ is the standard deviation of the FWHM distribution, and $\sigma_{registration}$ is the error in alignment of the Env and Gag fluorescent channels. In regions void of a Gag lattice ROI, Env tracks were classified to be distal. We quantified the differences in mobility between proximal and distal single Env trimers by first linking Env localizations based on proximity to one another and their frame interval (see Materials and Methods for track linking description). We observed marked differences in track appearance and shape between proximal and distal tracks, with distal tracks displaying random-walk or Brownian-like motion (Figure 4a).

Next, we computed the apparent diffusion coefficient ($D_{apparent}$) and slope of the moment scaling spectrum ($S_{MSS}$) for individual tracks, which mathematically describe the area covered by a single molecule in unit time and how well the molecule samples that area, respectively [43,44]. There was a significant shift in both $D_{apparent}$ and $S_{MSS}$ between proximal and distal tracks ($P_{D_{apparent}}=2.67\times 10^{-17}$, $P_{S_{MSS}}=1.04\times 10^{-15}$). $D_{apparent}$ for proximal tracks (0.042 μm${}^{2}$s${}^{-1}$) was roughly 40 percent lower than for distal tracks (0.068 μm${}^{2}$s${}^{-1}$), indicating that the Gag lattice acts as a diffusion barrier for the Env trimers when incorporated. There was also a 70 percent reduction in $S_{MSS}$ for proximal (0.028) versus distal tracks (0.099). Because the $S_{MSS}$ parameter indicates the shape or trajectory of a track as it samples an area of the membrane, a reduction in $S_{MSS}$ implies a more confined trajectory. An $S_{MSS}=0.028$ and $D_{apparent}=0.042$μm${}^{2}$s${}^{-1}$ for proximal Env concludes that the tracks are highly confined, with sub-viral diffusion over the time frame of acquisition (≈30 s). Quantification of these parameters suggests that Gag has a significant influence on Env during virus assembly compared to distal Env trimers on the plasma membrane (Figure 4).

The described methodology provides greater precision in the measurement of the Gag lattice perimeter, thus reducing the misclassification of proximal versus distal tracks and the associated error in diffusion measurements. Concurrently, our optimized BG18-QD625 probe enables tuneable high-density localization and tracking of single Env trimers on the surface of infected cells with tens of nanometers of precision.

## 3. Discussion

Many aspects of virus biogenesis remain enigmatic due to our inability to resolved the time-variant structural organization of viral sub-assemblies while simultaneously maintaining molecular specificity and physiologically relevant conditions. Time-resolved superresolution approaches, however, offer promise to further our understanding of the molecular steps and sequences of events that transpire during virus assembly. In this study, we present a method for sub-viral quantification of biophysical interactions between single molecules of HIV-1 envelope glycoproteins and the structural Gag lattice using live-cell single particle tracking and superresolution microscopy, respectively. This approach has allowed us to visualize nanoscale interactions between these molecules during virus biogenesis on the host-cell plasma membrane. We have shown that Gag lattices can be reconstructed to expected virus dimensions, below the diffraction-limit of the light microscope, using live-cell PALM. Further, we show that Env trimers can be simultaneously tracked and quantified based on their precise proximity to superresolved virus assembly sites.

Our approach has relied on the use of a genetically-encoded cytoplasmically-expressed nanobody fused to a reversibly switchable fluorescent protein to reconstruct virus assembly sites. The success of live-cell PALM is highly dependent on how well the sub-diffractive object is decorated with a fluorescent probe. We show that the CA-Skylan-S nanobody probe sufficiently samples the Gag molecules in a single virus assembly site because each superresolved image over a 30 s time course yields a FWHM approximately the size of expected virus assembly sites measured by electron microscopy [41]. The success of live-cell PALM imaging additionally requires sufficient sampling of each fluorescent probe within the structure of interest and high molecular brightness to reduce the uncertainty in the position of each probe. We judiciously selected the Skylan-S reversibly-switchable green fluorescent protein for its single molecule brightness and switching frequency. Skylan-S enabled us to sufficiently sample each nanobody tagged probe a few times before bleaching irreversibly, while also giving a sufficiently high photon count for each frame to more precisely localize single molecule probes. Our attempts with other variants of the Skylan series of reversibly-switchable green fluorescent proteins resulted in either low molecular brightness and poor localization precision or poor switching properties resulting in a lack of blinking and single molecule detection (data not shown). Finally, live-cell superresolution reconstructions require careful consideration of physical displacements of the object of interest, requiring tuning of sampling conditions to reduce motion blur artifacts [40,45]. In the case of HIV-1 Gag lattice formation, this structure remains sufficiently static over the sampling period (30 s) as evidenced by the FWHM reconstructions and expected diameters of HIV-1 particles.

Using our optimized fluorescent label and sampling conditions, we still, with minor frequency, found that our software filters out what is likely an assembly site that cannot be properly fit due to lack of localizations. Insufficient sampling at putative virus assembly sites, due to poor CA-Skylan-S probe labeling or sampling, may contribute to erroneous classifications of Env, resulting in an increase in apparent diffusion coefficient and $S_{MSS}$ distribution error. The sparsity of Env labeling with our methodology, however, statistically reduces the frequency of these false-negative events.

Additional considerations for implementation of this methodology are the production of a recombinant antibody fragment, subsequent site-specific unnatural amino acid modification and copper-free click chemistry to a quantum dot (Figure 2 and Materials and Methods). While unnatural amino acid constructs are publicly accessible and quantum dot kits are commercially available, production of the probe in usable quantities can be costly and technical for adopters without recombinant protein chemistry experience. The BG18-QD625 probe described here is a prime example of a highly optimized recombinant protein expression protocol and tuned conjugation chemistry, which yields large quantities of stable probe that can be used for months to years. Ultimately, live-cell superresolution and SPT methodology requires highly specific and high affinity (sub-10 nM dissociation constant) monovalent reagents for labeling of biological structures of interest due to the intrinsic single molecule sensitivity of these experiments.

The optimization of the CA-Skylan-S probe has provided a means for precise superresolution reconstructions of single HIV-1 assembly sites on the surface of live infected T-cells. Importantly, this resolution improvement has the potential to measure minor perturbations to Env mobility during lattice trapping. Further, our optimized production and validation of the BG18-QD625 probe has enabled us to tune the density of labeled Env trimers to maximize data collection on a per cell basis. This methodological advancement has provided insight into the biophysical interactions between the critical vaccine target HIV-1 Env and the underlying Gag lattice that coalesce to create an infectious virus particle. The sub-diffusive nature of Env in the Gag lattice could indicate a direct interaction between the membrane proximal matrix (MA) domain of Gag and the cytoplasmic tail of Env (Env-CT). We cannot rule out a physical or steric corralling mechanism, however, which creates a diffusion barrier to trap Env in a sub-diffusive state within the lattice. Further work will be aimed at elucidating potential molecular interactions using the methodology we have presented. Studies investigating diffusional behaviours of viral envelope glycoproteins with their respective structural components should lead to discovery of new therapeutic approaches that target virus assembly.

## 4. Materials and Methods

### 4.1. Reagents

The HEK293T (CRL-3216) cell lines were obtained from ATCC (Manassas, VA, USA). The CEM-A cell line was obtained through the NIH AIDS Reagent Program, Division of AIDS, NIAID, NIH: CEM-A from Dr. Mark Wainberg and Dr. James McMahon, CEM-CL10 [46]. Cell lines were grown at 37 ${}^{\circ }$C with 5% CO_2_. Complete growth medium for HEK293T cells was prepared by combining 10% fetal bovine serum (Corning; Corning, NY, USA), 2 mM L-glutamine (Corning), and Penicillin-Streptomycin (Corning) into Dulbecco’s Modified Eagle’s Medium (DMEM) (Corning) or for the CEM-A cell line with identical ingredients into Roswell Park Memorial Institute (RPMI) medium (Corning). Additionally, the CEM-A cell line growth medium was supplemented with 1% hypoxanthine, thymidine (HT) solution (Corning).

### 4.2. Generation of CA-Skylan-S Expressing Viruses

The pSV-NL4-3 reference HIV-1 genome was used for all experiments with constructs possessing deletions in the gag-p6 late-domain, *pol*, *vif*, *vpr*, and *nef* genes [6]. To create viral vectors coexpressing the CA-Skylan-S nanobody, the coding region for the anti-CA nanobody was genetically fused to the Skylan-S gene with a coding amino acid linker of: ‘RSFEFCSRRYRGPGIHRPVAT’. The CA-Skylan-S probe cassette was amplified with 5${}^{\prime }$ and 3${}^{\prime }$ Gibson homology arms against the coding regions of the 3${}^{\prime }$ end of *env* and 5${}^{\prime }$ end of the *nef* coding regions, respectively. Replication incompetent viruses harboring the CA-Skylan-S nanobody cassette in the *nef* splice site were produced by transfecting HEK293T cells with pSV-NL4-3 (described aboe) vector, pSPAX2 (Addgene, plasmid #12260), and pVSV-G (Addgene, plasmid #8454). Supernatants from cells expressing for 48 h were stored at −80 ${}^{\circ }$C prior to infection.

### 4.3. Monovalent BG18-QD625 Production

The BG18 anti-Env V3 glycan-binding light and heavy chain antibody sequences were obtained from the Protein Data Bank (6CH9) [47]. The fragment of antigen binding sequences were synthesized (IDT; Coralville, IA, USA) and cloned into the pCOMB3H-b12 vector using SacI and NotI sites, replacing the b12 fab coding sequence [48]. The pIII region of pCOMB3H was suppressed by introduction of a stop codon at the end of the BG18 heavy chain CH1 domain. The light chain gene fragment from BG18 was engineered to contain a 3${}^{\prime }$ amber stop codon (TAG) followed by a 6-fold repeat of histidine codons, finalized with an ochre stop codon (TAA). Periplasmic expression of BG18 fab containing a C-terminal p-azido-L-phenylalanine (pAZF) unnatural amino acid (Bachem; Torrance, CA, USA) was performed in *Escherichia Coli* XL1 Blue competent cells (Stratagene; San Diego, CA, USA) by co-transformation with pCOMB3H-BG18-LC-Amber-His6-Ochre and pEVOL-pAzF. Briefly, this strain was grown in 1–2 L of Super Broth medium containing selective antibiotics. At mid-log phase, 1 mM unnatural amino acid pAZF was added to the culture and expression of unnatural tRNAs and synthetase was induced with 2% (w/v) L-arabinose (Gold Biotechnology; St. Louis, MO, USA). Cellular lysates were produced by sonication in PBS pH 7.4 supplemented with 0.2 mM PMSF (Gold Biotechnology). Soluble proteins were clarified via centrifugation at 30,000× *g* for 30 min and filtration by 0.22 μm cellulose acetate syringe filter. BG18 fab that successfully suppressed the amber stop codon with pAZF was purified using Nickel affinity chromatography (Gold Biotechnology) followed by purification using CaptureSelect CH1-XL affinity chromatography resin (Thermo Scientific; Waltham, MA, USA). Elution fractions were pooled and dialyzed overnight in PBS, pH 7.4. Expression was assessed (Figure 2F) by SDS-PAGE analysis on 4–20% gradient gel (Bio-Rad; Hercules, CA, USA). The final yields from 1–2 L of culture were typically in the range of 1–3 mg of BG18-pAzF.

Copper-free click chemistry was performed between DIBO-QD625 (Site-Click kit, Thermo Scientific) and BG18-pAzF using a 1:1.5-fold stoichiometry, respectively. The reaction was allowed to proceed for 12–18 h at room temperature in the dark. Uncoupled DIBO-QD625 was removed from the reaction by another round of CaptureSelect CH1-XL affinity chromatography. Elution fractions were pooled and unreacted BG18-pAzF, lacking a DIBO-QD625 conjugate, was removed by filtration through a 100 KDa molecular weight cutoff filter (Site-Click kit) followed by repeated buffer exchanges with PBS, pH 7.4.

### 4.4. Sample Preparation and Imaging Conditions

CEM-A T-cells in complete RPMI medium were dispensed onto 25 mm No. 1.0 glass coverslips (Warner Instruments; Hamden, CT, USA) at high density and allowed to adhere for 18–24 h at 37 ${}^{\circ }$C and 5% CO_2_. Cells were infected with replication-incompetent (single-round infection) virus and incubated for 38–46 h prior to imaging. Coverslips were transferred to specimen holders and mounted on a custom-built ring-TIRF microscope with an environmental chamber maintaining 37 ${}^{\circ }$C and 5% CO_2_ previously described [27]. Briefly, BG18-QD625 and CA-Skylan-S probes were both excited with 473 nm laser light (50 mW at rear aperture) and fluorescence was detected by separating the respective wavelengths with a dichroic beamsplitter and W-View Gemini image splitter (Hamamatsu, Hamamatsu City, Japan). Images of each channel were then focused onto two halves of a liquid cooled ORCA Fusion scientific-CMOS camera (C14440-20UP). Images were streamed at 100 Hz.

### 4.5. Image Processing and Analysis

All imaging, single molecule localization, and channel registration was done per Pezeshkian et. al., 2019 [27]. Briefly, raw images were corrected for non-uniform pixel offset by use of a calibration map and were subsequently split based on wavelength. For CA-Skylan-S, frames with 10 ms exposure were integrated to 50 ms before single molecule localization and fitting using custom software (IDL, Harris Geospatial; Broomfield, CO, USA). A high density map of bead localizations using TetraSpeck fiducials (Thermo Fisher Scientific) was used to create a polywarp transform using the cp2tform function in Matlab (Mathworks, Natick, MA, USA) to correct for lateral chromatic aberration between the channels. Single molecule localizations were then handed to an automated package written in MATLAB. CA-Skylan-S localizations with precision below 40 nm were used to reconstruct a 50 nm pixel size binned image. This image was sent through a cluster finding algorithm which extracted each Gag cluster centroid (window 150 × 150 nm) using a threshold that requires a minimum number of localizations and integrated probability density above 1 standard deviation from the background. Single molecule localizations of Env were then deemed proximal or distal to a Gag lattice by use of Equation (1). The separated localizations were then linked using a previously described track linking algorithm adapted to MATLAB from Crocker and Grier 1996 [49]. The parameters for linking are as follows: 50 ms time gap, 10 minimum localizations per track, 1 pixel (108.33 nm) maximum frame to frame distance. The remaining tracks were analyzed as previously described by using the moment scaling spectrum to calculate $D_{apparent}$ and $S_{MSS}$ [27,44]. Mean squared displacement (MSD) was fit to a linear regression solving for $D_{apparent}$ using the lag time, τ (Equation (2)).

(2) $$MSD=4D_{apparent}\tau^{\alpha }$$

Localization uncertainties were then used to weight the MSD fits (Equation (3)), where $R^{o}$ is displacement and $\sigma_{R}$ is standard deviation.

(3) $$\frac{\Sigma \sigma_{R}R^{o}}{\Sigma (\sigma_{R})}$$

### 4.6. Monte Carlo Simulations of Single Molecule Localization and HIV-1 Assembly Site Reconstruction

Simulations were performed using custom scripts written in MATLAB. Simulations were designed to assess the accuracy of reconstructions and centroid finding for scenarios with differing numbers of CA-Skylan-S probes per lattice and variances in molecular reappearance. CA-Skylan-S molecules were semi-randomly distributed on the surface of a sphere with a radius of 75 nm (Figure S3). Our simulations constrained the molecular positions to discontinuous regions between the polar angles ($\frac{5\pi }{3},\frac{\pi }{3}$) and ($\frac{2\pi }{3},\frac{4\pi }{3}$) to mimic the open neck of the viral bud and the increased accessibility of equatorial CA binding sites, respectively. True molecular positions were then shifted pseudo-randomly to mimic centroid uncertainty. This pseudo-random displacement vector was selected from a normal distribution centered around its position with a standard deviation of 50 nm, approximately the sum of our maximum localization uncertainty and standard deviation of our localization precision in experimental data ($\sigma_{max}=40\pm 9$ nm). Each simulation used a fixed number of molecular reappearances (α= 1 to 5) resulting in total localizations (1 to 50) to assess the accuracy of reconstruction. For each condition, 50 simulated assembly site X/Y-FWHM and centroids were averaged for each value of α.

## Supplementary Materials

The following are available online athttps://www.mdpi.com/2076-0817/9/11/972/s1, Figure S1: Distribution of Skylan-S localizations at single virus assembly sites. Figure S2: Full-width at half-maximum (FWHM) anisotropy in X- and Y-dimensions. Figure S3: Simulations of localizations and reappearances required for reconstruction and centroid finding of assembly sites. Figure S4: Fluorescence crosstalk of Skylan-S is insignificant and does not lead to localization artifacts in the QD625 channel. Figure S5: Fluorescence intensity of Immobilized QD625 remains relatively constant over relevant acquisition times and excitation intensities.

## Abbreviations

The following abbreviations are used in this manuscript:

Env	Envelope
MA	Matrix
CA	Capsid
SPT	Single Particle Tracking
MSD	Mean Squared Displacement
$S_{MSS}$	Slope of the Moment Scaling Spectrum
TIRF-M	Total Internal Reflection Fluorescence Microscopy
