# Supplementary material for: A Quantitative Live-Cell Superresolution Imaging Framework for Measuring the Mobility of Single Molecules at Sites of Virus Assembly
Source: Pathogens. 2020 Nov 21;9(11):972. doi: 10.3390/pathogens9110972 (PMC7700196; doi:10.3390/pathogens9110972)
Supplement: Supplementary file 1 [file pathogens-09-00972-s001.pdf]

Supplementary Materials: A Quantitative Live-Cell Superresolution Imaging Framework for Measuring the Mobility of Single Molecules At Sites of Virus Assembly

Groves, S, Nicholas [1], Bruns, Merissa M. [1] and van Engelenburg, Schuyler B. [1,*],

1. Supporting Information

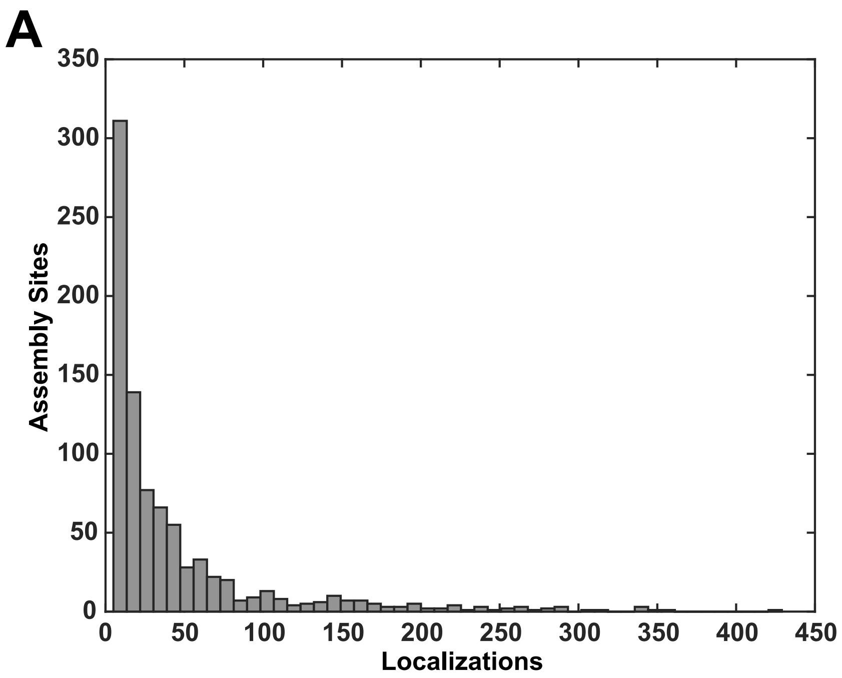

Figure S1. Distribution of Skylan-S localizations at single virus assembly sites. **(A)** The mean number of localizations for the ensemble of assembly sites measured was determined by fitting to a lognormal distribution. On average, 23 localizations were found at assembly sites after filtering out localizations with uncertainties below 40 nm and eliminating assembly sites with less than 5 localizations.

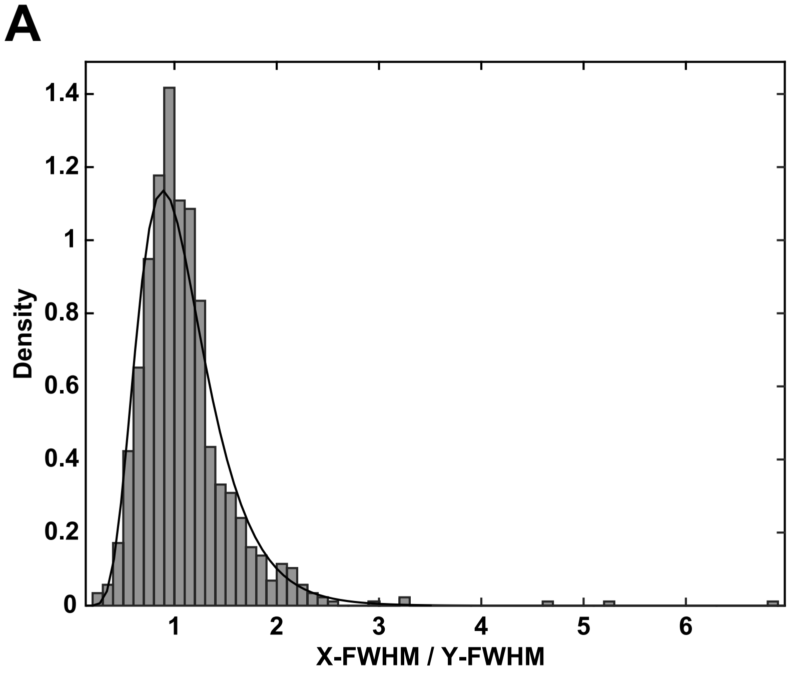

Figure S2. Full-width at half-maximum (FWHM) anisotropy in X- and Y-dimensions. **(A)** Pairwise FWHM anisotropy was calculated for all independent assembly sites by the equation $\frac{\text{X-FWHM}}{\text{Y-FWHM}}$. This fractional FWHM was fit to a lognormal distribution, with mean $\mu = 1.09$ and standard deviation equal to $\approx$ 4% of X- and Y-FWHM measurements. This slight anisotropy results in approximately 5.7 nm of uncertainty in the centroid position, only 17% of our mean localization precision.

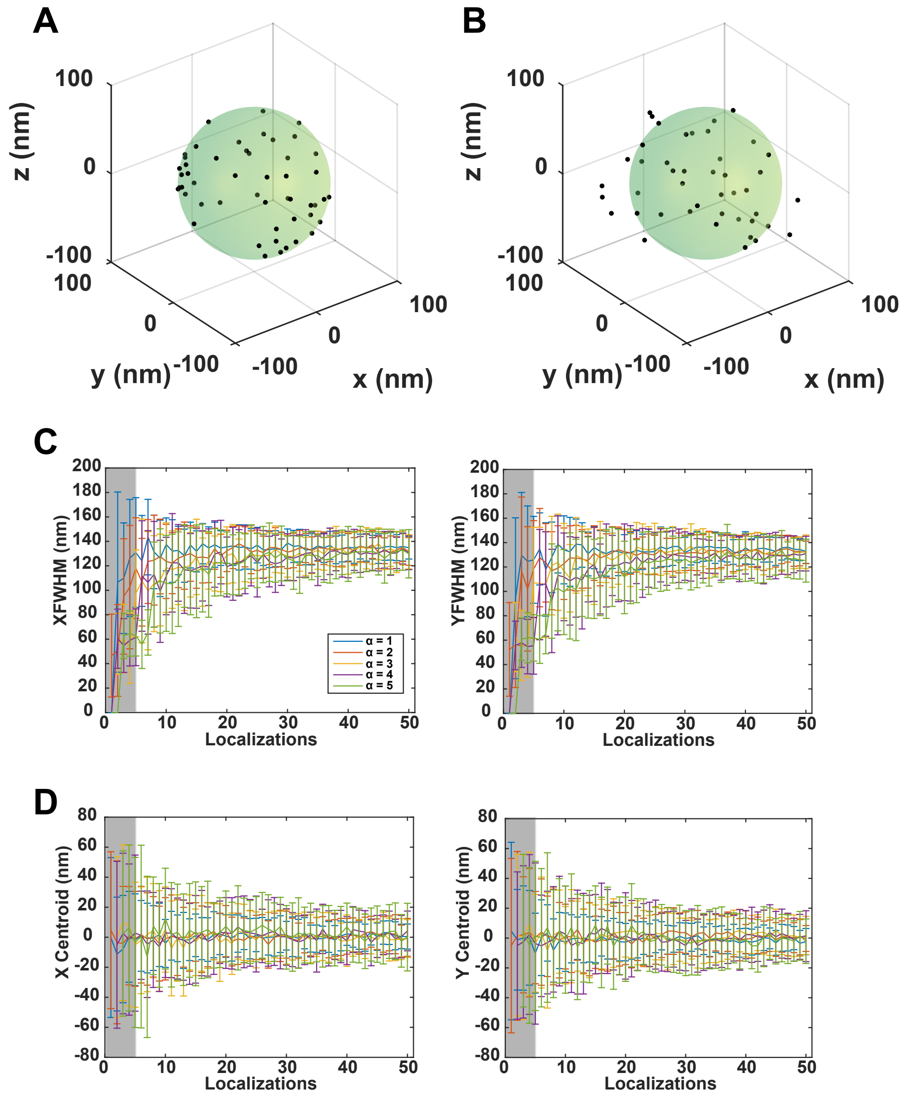

Figure S3. Simulations of localizations and reappearances required for reconstruction and centroid finding of assembly sites. **(A)** Using a sphere constrained by the discontinuous polar angle ranges $(\frac{5\pi}{3}, \frac{\pi}{3})$ and $(\frac{2\pi}{3}, \frac{4\pi}{3})$, we simulated arrangements of pseudo-random molecules labeling a Gag shell. These constraints were selected because the CA-Skylan-S probe is unable to label a budding lattice at the bottom most region of the assembly site since the neck is open. Further, it is likely that the CA-Skylan-S probe labels the crown of the lattice with low frequency, since there are proximal Gag molecules near the cytosolic entrance at the neck and equator of the assembly site. **(B)** Localizations are then pseudo-randomly displaced to mimic localization uncertainty measured in this study. **(C)** Reconstructions of X- and Y-FWHM approach the experimentally determined mean FWHM of ≈138 nm for all degrees of molecular reappearances (α). **(D)** The X and Y centroids describe the lateral origins of the Gag sphere with accuracy at or below the localization uncertainty. Gray regions indicate the localization cutoff used in this study (5 localizations) for reconstructing bona-fide assembly sites with the CA-Skylan-S probe. Error bars represent standard deviation. These simulations suggest that our experimental system estimates the size and centroid of virus assembly sites through localization of more than one CA-Skylan-S molecule labeling the Gag lattice, with these probes likely exhibiting more than one molecular reappearance.

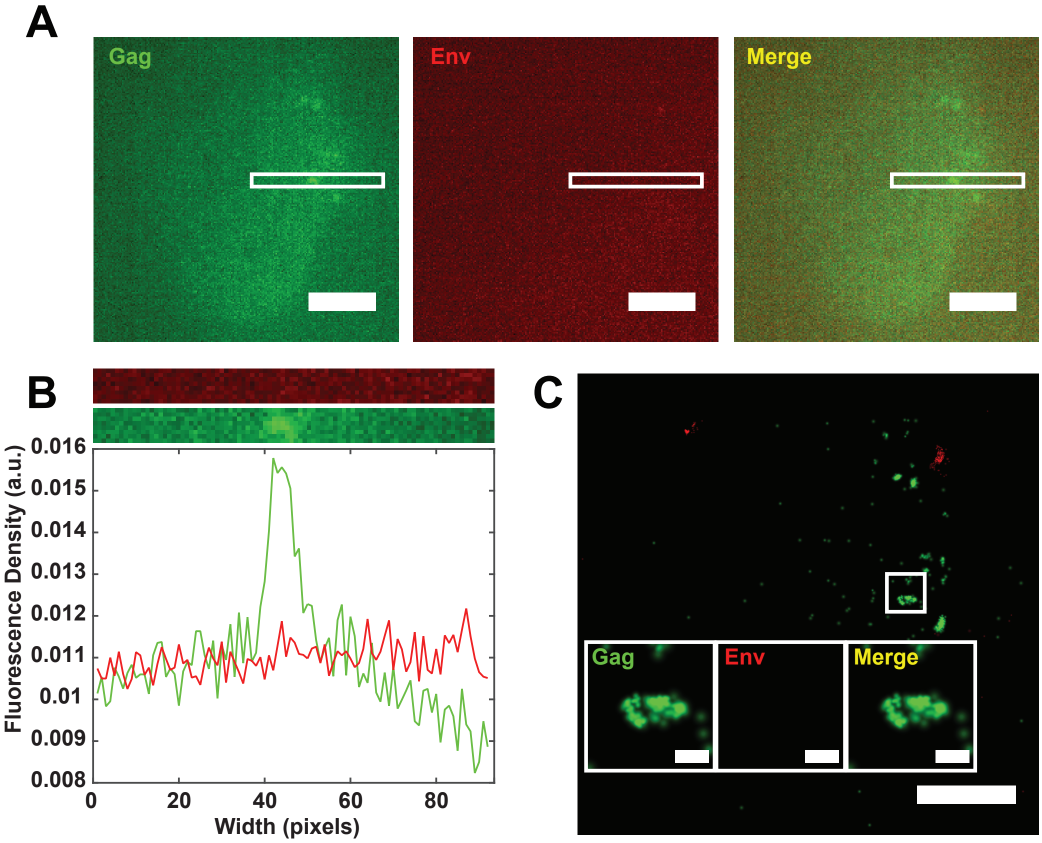

Figure S4. Fluorescence crosstalk of Skylan-S is insignificant and does not lead to localization artifacts in the QD625 channel. **(A)** Single images for CA-Skylan-S labelling of the Gag lattice (Gag, 50 ms integration) and BG18-QD625 (Env, 10 ms integration). White box highlights a region of high signal intensity for the CA-Skylan-S channel (scale bars = 5 μm). **(B)** The fluorescence density of the white bounded region of interest in **A**. No appreciable signal is observed in the BG18-QD625 channel compared to the CA-Skylan-S channel in this region of interest. **(C)** Point localization reconstructions of the field of view in **A**. Insets highlight the peak signal intensity in **B**. In this region void of true BG18-QD625 signal, no point localizations are detected by the peak finding algorithm (scale bar = 5 μm, inset scale bar = 500 nm.)

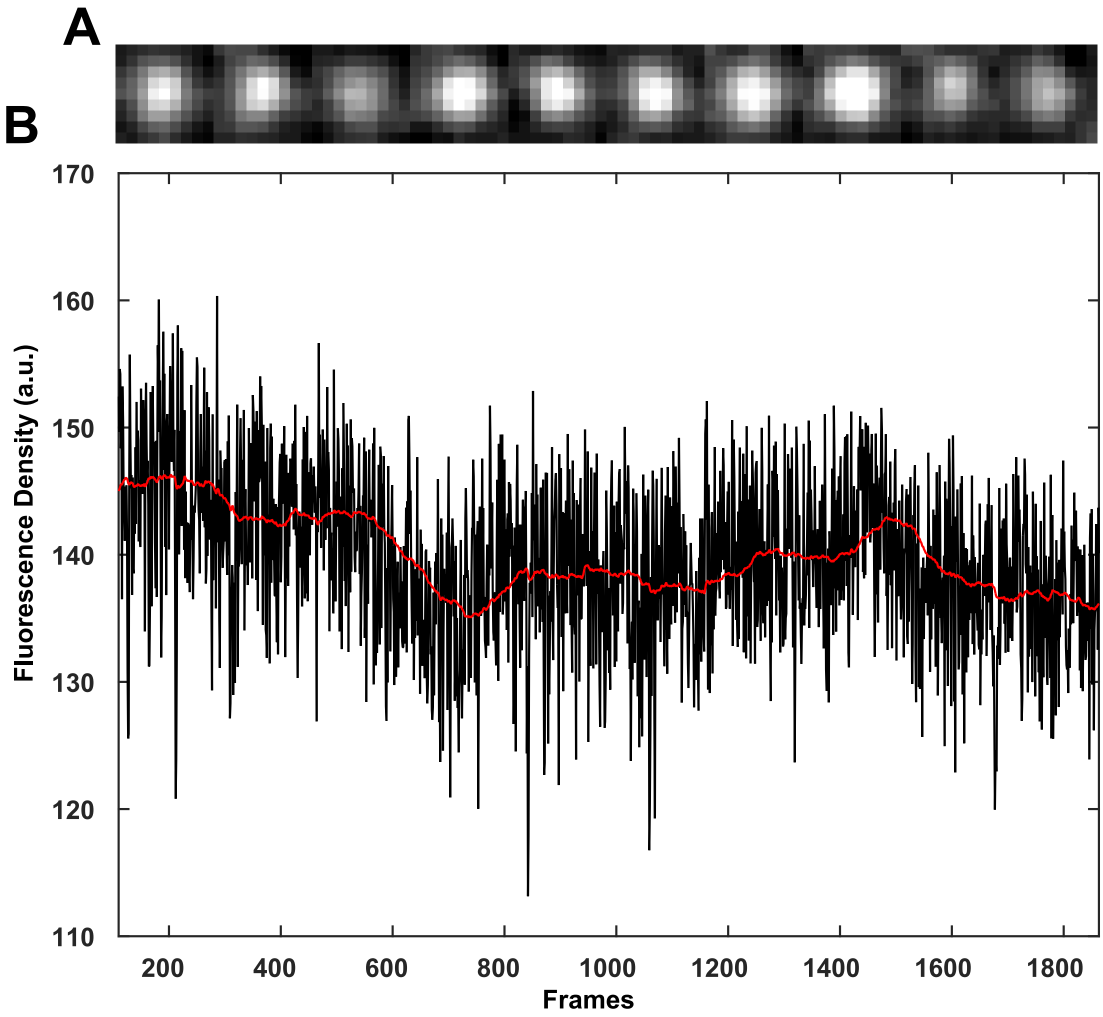

Figure S5. Fluorescence intensity of Immobilized QD625 remains relatively constant over relevant acquisition times and excitation intensities. **(A)** Montage of QD625 evenly sampled over the acquisition period graphed in **B** (10 ms integration). **(B)** Fluorescence density from the 9×9 pixel area defining static QD625 in **A** (n = 1753 image frames, Black: Fluorescence Density (a.u.) of individual frames, Red: 100 frame window moving average). Fluorescence fluctuations are present, however no appreciable photobleaching or dimming is detected (5 percent decrease in fluorescence density over 17.53 seconds). Fluctuations in intensity are likely due to stochastic fluctuations in excitation intensity, fluorescence transitions, and camera noise.
